# Life-history traits predict perennial species response to fire in a desert ecosystem

**DOI:** 10.1002/ece3.1159

**Published:** 2014-07-10

**Authors:** Daniel F Shryock, Lesley A DeFalco, Todd C Esque

**Affiliations:** 1 U.S. Geological Survey, Western Ecological Research Center160 N. Stephanie St., Henderson, Nevada

**Keywords:** Fire, Mojave Desert, plant functional types, trait analysis

## Abstract

The Mojave Desert of North America has become fire-prone in recent decades due to invasive annual grasses that fuel wildfires following years of high rainfall. Perennial species are poorly adapted to fire in this system, and post-fire shifts in species composition have been substantial but variable across community types. To generalize across a range of conditions, we investigated whether simple life-history traits could predict how species responded to fire. Further, we classified species into plant functional types (PFTs) based on combinations of life-history traits and evaluated whether these groups exhibited a consistent fire-response. Six life-history traits varied significantly between burned and unburned areas in short (up to 4 years) or long-term (up to 52 years) post-fire datasets, including growth form, lifespan, seed size, seed dispersal, height, and leaf longevity. Forbs and grasses consistently increased in abundance after fire, while cacti were reduced and woody species exhibited a variable response. Woody species were classified into three PFTs based on combinations of life-history traits. Species in Group 1 increased in abundance after fire and were characterized by short lifespans, small, wind-dispersed seeds, low height, and deciduous leaves. Species in Group 2 were reduced by fire and distinguished from Group 1 by longer lifespans and evergreen leaves. Group 3 species, which also decreased after fire, were characterized by long lifespans, large non-wind dispersed seeds, and taller heights. Our results show that PFTs based on life-history traits can reliably predict the responses of most species to fire in the Mojave Desert. Dominant, long-lived species of this region possess a combination of traits limiting their ability to recover, presenting a clear example of how a novel disturbance regime may shift selective environmental pressures to favor alternative life-history strategies.

## Funding Information

U.S. Bureau of Land Management (BLM)

## Introduction

Fire acts as an evolutionary filter in vegetation communities, favoring species with traits that allow them to survive burn injury or to successfully reproduce in postfire environments (Bond and Keeley 2005; Pausas et al. 2006). Consequently, fire-prone vegetation communities are composed of species capable of regenerating through a variety of mechanisms, including resprouting, serotiny, and smoke-induced germination (Bond and van Wilgen 1996; Allen 2008). However, where fire is a novel disturbance – for example, when facilitated by invasions of non-native species – consequences can be dramatic, including transitions to alternative community states (Mack et al. 2000; Brooks et al. 2004).

Ecological theory predicts that trait convergence should occur in communities subjected to a novel fire regime, as species with traits limiting their ability to cope with the altered postfire environment are removed (Keddy 1992; Pausas and Verdu 2008; Moretti and Legg 2009). However, species composition of burned communities may simultaneously diverge due to variation in initial community composition and postfire environmental conditions (Fukami et al. 2005; Smart et al. 2006), complicating efforts to predict species-level changes through time. Under these circumstances, a plant functional type (PFT) approach may be particularly well suited for modeling community dynamics, where PFT is defined as a group of species with a similar response to disturbance based on a shared combination of functional traits (Lavorel and Garnier 2002). By generalizing beyond specific species associations and localities, PFT approaches have provided accurate, broad-scale predictions of vegetation response to a variety of conditions, including fire (Pausas et al. 2004; Keith et al. 2007; Gosper et al. 2012) and other disturbances (McIntyre and Lavorel 2001; De Bello et al. 2005).

The Mojave Desert of the southwestern United States is a clear example of a region experiencing widespread ecological change in response to a novel fire regime (Brooks and Minnich 2006). Over the past several decades, this region has experienced an unprecedented frequency of large-scale wildfires, fueled by invasive annual grasses in a process known as the invasive grass/fire cycle (D'Antonio and Vitousek 1992; Brooks and Esque 2002; Brooks and Matchett 2006). In 2005 alone, nearly one million acres within the Mojave Desert burned – an area larger in size than all fires in the previous 25 years combined (Brooks and Matchett 2006). Given that Mojave Desert vegetation has only recently become fire-prone (Brooks and Minnich 2006), many perennial plant species lack adaptations for regeneration after fire, and the resulting shifts in species composition and diversity have been severe, particularly in areas burned more than once (Steers 2008; Brooks 2012). However, the nature and duration of compositional change has varied based on initial community types (Engel and Abella 2011), and managers have grappled with how best to prioritize and plan restoration efforts. A PFT-based model of postfire vegetation change in this system would provide at least three advantages, including: (1) the ability to predict the responses of a large number of species to fire; (2) the ability to generalize predictions across community types; and (3) the ability to determine which communities will be slowest to recover, based on their PFT composition.

In adopting a PFT approach, two key conditions should be met to retain ecological relevance: (1) the approach should be based on a model of vegetation assembly appropriate for the system under study and (2) functional traits used to group species should be relevant to the key successional processes identified by the underlying assembly model (Keith et al. 2007; Gosper et al. 2012). In fire-prone environments, studies have found support for Egler's (1954) initial floristic composition (IFC) model, which stipulates that most species present during a successional sequence re-establish shortly after disturbance, with minimal dispersal and subsequent establishment of new species (Wilson et al. 1992; Barbour et al. 1999). Subsequent shifts in species composition reflect the differential growth rates, competitive abilities, and longevities of component species. Evidence for the applicability of this model to postfire succession in the Mojave Desert includes monotonic declines in diversity and cover following multiple burns (Steers 2008; Brooks 2012), and the fact that most species present in disturbed areas are also components of undisturbed vegetation, though in different quantities (Webb et al. 1988; Abella 2009).

Adopting IFC as a means of explaining postfire vegetation assembly requires identifying which plant traits influence postfire persistence or establishment, along with those related to growth, competitive ability, and longevity. In terms of persistence and establishment, studies in fire-prone environments generally group species according to a dichotomy between resprouters and seeders (Pausas et al. 2004; Vesk et al. 2004). However, the proportion of species resprouting after fire is greater at the upper end of precipitation and productivity gradients, while obligate seeders are more abundant in areas with low precipitation (Clarke et al. 2005; Pausas and Bradstock 2007; Nano and Clarke 2011). In the Mojave Desert, where average annual precipitation ranges from 50 to 130 mm in valley bottoms (Redmond 2009), resprouting is physiologically limited and dependent upon fire intensity and postfire climatic conditions (Brooks and Minnich 2006; Abella 2009; DeFalco et al. 2010). As a result, recovery for most perennial species occurs predominantly through seedling recruitment, and seed-related traits are therefore likely to be better predictors of postfire establishment than resprouting ability for most Mojave Desert species.

Methods for quantifying differences in relative growth rate, competitiveness, and longevity are now well established in life-history theory. It is widely accepted that fundamental resource allocation tradeoffs result in life-history strategies with characteristic functional traits and demographic patterns (e.g., Diaz and Cabido 1997; Barbour et al. 1999). One example is the tradeoff between investment in reproduction and investment in survival, which is reflected by differences in lifespan and seed size (Franco and Silvertown 2004; Moles and Westoby 2006; Adler et al. 2014). Growing empirical evidence suggests that simple morphological traits can predict life-history strategies of species across different regions (Diaz et al. 2004; Adler et al. 2014), and core lists have been proposed relating many of these traits to disturbance response (Weiher et al. 1999). However, tests for the ability of such traits to form the basis of PFT approaches predicting community response to disturbance are still lacking across a broad range of ecosystems.

Beginning with the premise that postfire vegetation recovery in the Mojave Desert follows patterns underlain by the IFC model, we test the hypothesis that life-history traits can predict perennial species response to fire in this environment. Further, we address the complementary hypothesis that species can be grouped into PFTs with a consistent response to fire based on natural co-occurrences of these traits. To evaluate these complementary hypotheses, we compiled datasets reflecting vegetation response to fire in the Mojave Desert at both short- and longer-term time scales and across a broad regional gradient. Results from this analysis will have direct applied relevance for land managers in our study system while addressing the question of whether PFTs based on simple morphological traits can predict perennial species response to fire in a desert environment that was historically not fire-prone. In an era of global change, the ability of PFTs to predict ecosystem response to novel disturbance regimes is a recurring issue in global vegetation modeling (Diaz and Cabido 1997), and whether or not PFT approaches can be based on easily observed traits is a central question due to the limited availability of trait information for many species (Cornelissen et al. 2003; Diaz et al. 2004; Lavorel et al. 2007).

## Materials and Methods

### Analysis framework

Our analysis proceeded in four steps aimed at identifying life-history traits that influence perennial plant species recovery following wildfire and then determining whether species could be grouped into PFTs based on these traits. In the first step, we compiled life-history trait data for all perennial plant species included in short-term (1–4 years) and long-term (up to 52 years) postfire vegetation datasets. Second, we quantified individual species recovery following wildfire using a constrained ordination procedure. Third, we identified those life-history traits with a significant influence on species recovery through multivariate permutation testing. Finally, we derived and evaluated fire-response PFTs through a classification of species based on significant life-history traits. Each stage of our analysis is described in greater detail below. Species nomenclature throughout this study follows Baldwin et al. (2012).

### Step 1: Compilation of vegetation and trait data

#### Short-term dataset

Perennial species cover was measured at four sites from 1 to 4 years following wildfires in 1993 or 1999 on burned and adjacent unburned areas (serving as controls) in the northeast Mojave Desert (see Table 1 for additional details). Average (± SD) annual precipitation at Littlefield, Arizona (closest to the Littlefield, Bulldog, and Jump Creek transects, Table 1), is approximately 186 ± 70 mm, while at St. George, Utah (closest to the Mill Creek transects, Table 1), is approximately 208 ± 54 mm (Western Regional Climate Center, www.wrcc.dri.edu). All live perennial plant cover was measured along randomly located transects using a line-intercept technique, in which the canopy of all plants intersecting a transect line was measured. Vegetation cover measurements from all four sites were combined to form the short-term dataset with a total of 685 transects and 35 species. Fire treatment (burned or unburned) and the number of years postfire (1–4) were also recorded individually for each transect. Consistent with our aim of generalizing species responses across a range of conditions, this dataset included measurements conducted in several different vegetation communities (Table 1).

**Table 1:** Perennial cover data collected after fires in the northeast Mojave Desert (USGS, Western Ecological Research Center, Las Vegas Field Office). All transects were sampled using line-intercept. UTMs use NAD83 datum, zone 12 N. B/U = burned/unburned.

Site	UTMs	Community dominants^1^	Perennial cover (%)^2^	Fire	Years measured	Transect length	# Transects (B/U)
Littlefield, AZ	4098328 E 0244093 N	LATR-AMDU	23	1999	2000–03	20 m	30/30
Jump Canyon, AZ	4056522 N 0255702 E	JUOS	36	1999	2000–03	50 m	10/10
Jump Canyon, AZ	4054634 N 0260362 E	CORA	48	1999	2000–03	50 m	5/5
Jump Canyon, AZ	4057573 N 0258977 E	ARTR	47	1999	2000–03	50 m	5/5
Bulldog, UT	4100840 N 0242970 E	CORA	25	1993	1993–97	50 m	10/10
Mill Creek, UT	4116236 N 0242970 E	LATR-AMDU	18	1993	1993, 95–97	50 m	33/33

1 LATR-AMDU (*Larrea tridentata-Ambrosia dumosa*); JUOS (*Juniperus osteosperma*); blackbrush (*Coleogyne ramossisima*); and sagebrush (*Artemesia tridentata*).

2 Average cover of live perennial species measured along unburned line-intercept transects.

#### Long-term dataset

To derive a long-term dataset of perennial species response to fire, we combined cover and density values reported from six published studies of postfire perennial vegetation in the Mojave Desert, including five studies reporting foliar cover values (Callison et al.^1985^; Brown and Minnich 1986; Minnich 1995; Webb et al. 2003; Engel and Abella 2011) and one study reporting plant density (Steers 2008). Studies reporting cover values differed in sampling technique, with three reporting line-intercept measurements and two reporting visual cover-class estimates from quadrats. In order to account for measurement differences between studies, we relativized the combined plot × species matrix by dividing each row in the matrix by the sum of all elements in that row, creating a measure of relative abundance. Because all plots had unburned controls, later analysis (step 2, below) reflected differences in relative abundance between burned and unburned areas regardless of measurement technique. The combined matrix included 118 plots and 49 perennial species and spanned a time period ranging from 1 to 52 years postfire.

The six studies represented a wide geographic area, including southwestern Utah (Callison et al. 1985), southern Nevada (Webb et al. 2003; Engel and Abella 2011), and southeastern California (Brown and Minnich 1986; Minnich 1995; Steers 2008). There was some geographic overlap among fires sampled by Brown and Minnich (1986) and Steers (2008); however, sampling for these studies was conducted during different decades and in separate plots. The six studies also spanned several vegetation community types, including creosote bush (*Larrea tridentata*) scrub, blackbrush (*Coleogyne ramosissima*) scrub, and Joshua tree (*Yucca brevifolia*) woodland (MacMahon 2000; West and Young 2000). This dataset represented much of the available quantitative information concerning vegetation response to wildfire in the Mojave Desert and thereby enabled detection of broad-scale patterns relating to species life histories across different vegetation associations.

#### Species traits

We selected eight life-history traits thought to influence vegetation response to disturbance based on reviews of this topic (e.g., McIntyre et al. 1999; Lavorel and Garnier 2002; Cornelissen et al. 2003; Lavorel et al. 2007). The traits, their definitions, and hypothesized ecological functions are provided in Table 2. These traits also represent the different processes influencing vegetation succession under the IFC model – persistence/regeneration, competitive ability, growth rate, and longevity – by distinguishing different life-history strategies. Lifespan and seed size distinguish species with fast life histories and high fecundity from those with slow life histories and high survival (Franco and Silvertown 2004; Adler et al. 2014), while short leaf lifespans indicate large investment toward growth (Reich et al. 1992; Adler et al. 2014). Seed dispersal relates to regeneration ability (Noble and Slatyer 1980; McIntyre et al. 1999), while maximum height and vegetative spread may both serve as indicators of competitive ability (Weiher et al. 1999; Keith et al. 2007). Growth form and growth structure both relate to the resistance and resilience of a species to physical damage, thereby influencing the level of persistence. However, growth form influences a variety of other factors, including growth rate and generation time (Silvertown et al. 1993), and is regarded as one of the major determinants of a species' ability to cope with disturbance (Lavorel et al. 1997).

**Table 2:** Plant traits selected for inclusion in analysis of Mojave Desert perennial species response to wildfire. Variable coding in the second column of the table describes the manner in which species' trait values were input to ordination and classification procedures. Full trait values for all species are provided in Appendix S1.

Trait	Definition	Ecological function
Growth form	Graminoid, forb, cactus, woody	Persistence, regeneration, growth rate
Growth structure	1 = Prostrate, 2 = Erect, 3 = Erect with multiple stems	Persistence
Height (m)	1 = <0.5, 2 = 0.5–1, 3 = 1–1.5, 4 = 1.5–2, 5 = 2–4, 6 = > 4	Competitive ability
Leaf longevity	E = Evergreen, CD = Winter deciduous, DD = Drought deciduous	Growth rate
Lifespan (years)	1 = Short (1–20), 2 = Moderate (20–100), 3 = Long (>100)	Regeneration, growth rate, longevity
Seed size (mg)	1 = Small (<1), 2 = Medium (1–5), 3 = Large (>5)	Regeneration
Seed dispersal	0 = Not wind dispersed, 1 = Wind dispersed	Regeneration
Vegetative spread	0 = None, 1 = Clonal growth by stem splitting, 2 = Clonal growth by rhizomes	Competitive ability

Values for each trait were assigned to species based on a combination of literature sources and databases (Table 2, Table S1). Growth form and growth structure were defined according to Baldwin et al. (2012) or the flora of North America (http://floranorthamerica.org/). Species heights were defined using the maximum value from ranges provided in Baldwin et al. (2012). Seed mass values were taken from the Kew Seed Information Database (Royal Botanic Gardens Kew 2014, http://data.kew.org/sid/) and the Baker Seed Herbarium (Jepson Flora Project 2006, http://ucjeps.berkeley.edu/EFT.html). Both height and seed mass were also split into ordinal classes, rather than treated as continuous variables, in order to minimize the potential for error in trait assignments (Table 2). Because many seeds are dispersed by ants and rodents in deserts in general (Gutterman 1994) and in the Mojave Desert in particular (DeFalco et al. 2009, 2012), we included seed dispersal as a binary trait separating wind-dispersed from non-wind-dispersed species. Species were classified as wind-dispersed if their seeds possessed mechanisms to aid in dispersal, including wings, hairs, or small size (<1 mg), and did not possess obvious mechanisms for zoochory (e.g., burs, encased in fleshy fruit). Finally, we defined three broad classes for the lifespan trait (Table 2) and based estimates on observational or empirical studies (e.g., Bowers et al. 1995) or information from databases (USDA Fire Effects Information System, http://www.feis-crs.org/beta). All perennial forbs were classified as short-lived (1–20 years). Full trait values, along with source information, are provided in Appendix S1.

### Step 2: Species recovery following fire

In order to quantify species responses to fire through time, we applied the outlying mean index (OMI) constrained ordination to both datasets (Doledec et al. 2000). OMI does not assume any particular mathematical distribution in species' responses to the environment and gives equal weight to species-poor and species-rich sites (Kleyer et al. 2012). In the resulting ordinations, OMI places species (columns of the input matrix) in environmental space rather than sites (rows). In biological terms, this means that species with a high score on an OMI axis are exhibiting a strong response to the environmental variables used as axis constraints, whereas species located near the origin are exhibiting a weak response (i.e., generalist species). We used fire treatment (burned or unburned) as a constraining factor in OMI ordinations for both datasets. The number of years postfire (1–4 years) was also used as a constraining variable for the short-term dataset, while, for the long-term dataset, we grouped plots into postfire decade (1–5 decades). After conducting OMI on both datasets, we extracted species scores (positions of species on *x*-and *y*-axes of ordination) for use as response variables in step 3. These values reflect only the variability in the input datasets associated with the two constraining variables (burn treatment and years or decades postfire) and therefore represent a relative measure of species abundances in burned versus unburned areas through time.

### Step 3: Identification of significant traits

Using OMI species scores from the two constrained axes as response variables reflecting species recovery following wildfire, we then evaluated whether life-history traits exerted a significant influence on these values. Species traits were used as explanatory variables against OMI species scores as response in distance-based permutational multivariate analysis of variance (perMANOVA; Anderson 2001). Individual perMANOVA tests (10,000 permutations with Euclidean distance) were conducted for each trait (Table 2) against species scores on the two OMI axes. All testing was performed separately on the short- and long-term datasets, allowing a comparison of the importance of traits over different time scales. Additionally, for all traits except growth form and growth structure, we conducted separate tests including all species and for woody species alone (those with developed periderm).

### Step 4: Derivation and evaluation of functional groups

Traits found to explain a significant (*P* ≤ 0.05) amount of variability in OMI species scores for woody species were then included in a hierarchical agglomerative cluster analysis of this growth form, including all woody species found in the short- and long-term datasets. We chose not to include perennial grasses, forbs, and cacti in the classification for two reasons: (1) because these species already responded consistently to fire (e.g., grasses and forbs typically increased; cacti decreased) and (2) because classifications based on morphology should be conducted separately for different growth forms (Lavorel et al. 1997), but there was not enough replication of grasses, forbs, or cacti in either the short- or long-term datasets to compare the fire responses of different subgroups. In contrast, woody species were highly variable in their response to fire and had adequate replication in our datasets (*n* = 36).

Cluster analysis was performed using Gower's distance measure, which can accommodate both ordinal and categorical variables (Gower 1971; Kaufman and Rousseeuw 1990) and Ward's minimum-variance linkage method. PFTs for woody species were then derived using the dendrogram that resulted from the cluster analysis. The growth form categories of grasses, forbs, and cacti were also treated as separate PFTs, and the full set of PFTs was then evaluated for its ability to predict species responses to fire using the same methods as in step 3 (above). Specifically, we fit perMANOVA models separately to each dataset using OMI species scores as response variables and PFT group (including grass, forb, and cacti categories) as an explanatory factor.

### Statistical procedures

All statistical procedures were carried out using R statistical software, version 3.0.0 (R Development Core Team 2013). OMI ordinations were performed using the “dudi.hillsmith” and “niche” functions in the R package “ade4” (Dray and Dufour 2007), while perMANOVA was carried out using the “adonis” function in the R package “vegan” (http://Cran.r-project.org/package=vegan). Finally, hierarchical clustering was performed using the “daisy” and “agnes” functions in the R base package “cluster” (http://cran.r-project.org/web/packages/cluster/index.html).

## Results

### Species recovery following fire

**Figure 1:**
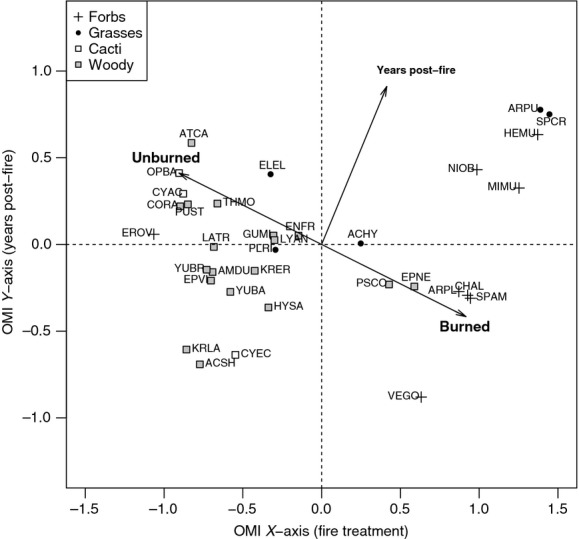
Biplot showing outlying mean index ordination of 35 species in the short-term data set. Ordination axes were constrained by fire treatment (burned or unburned) and number of years postfire (1–4). The length and direction of arrows indicates the degree to which the ordination is structured by variability associated with the constraining variables. Full scientific names for species are provided in Appendix S1.

The OMI ordination of the short-term dataset explained a significant proportion of variability in species composition (*P* < 0.001), indicating strong concordance with the constraining variables. Slightly greater than 90% of the constrained variability could be attributed to the OMI *X*-axis, which corresponded to fire treatment based on the biplot (Fig. 1). Species with high scores on this axis increased in abundance in burned areas relative to unburned controls, while species with lower scores decreased. In terms of growth form, the majority of perennial grass and forb species present at the study sites scored positively on the OMI *X*-axis (11/14 species). Of the woody species, only *Ephedra nevadensis* and *Psilostrophe cooperi* scored positively, while every cactus species scored negatively. The small amount of constrained variability attributed to the OMI *Y*-axis (<10%), relating to postfire year, suggests that postfire vegetation changes were minimal at these sites after 4 years.

**Figure 2:**
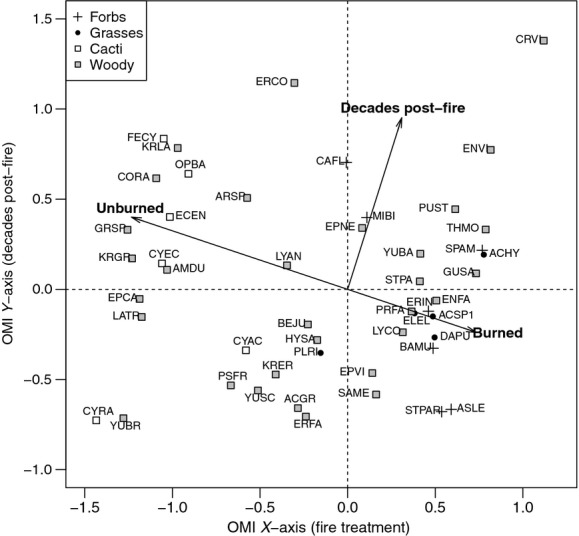
Biplot showing outlying mean index ordination of 49 species in the long-term dataset. Ordination axes were constrained by fire treatment (burned or unburned) and number of decades (1–5 decade) postfire. The length and direction of arrows indicates the degree to which the ordination is structured by variability associated with the constraining variables. Full scientific names for species are provided in Appendix S1.

The OMI ordination of the long-term dataset also explained a significant proportion of variability in species composition (*P* < 0.001), with 77% of the constrained variability attributed to the OMI *X*-axis, again corresponding to fire treatment (Fig. 2). As with the short-term dataset, species with positive scores on the OMI *X*-axis increased in abundance following fire, while species with negative scores decreased. The larger amount of variability associated with the OMI *Y*-axis (23%) compared with that of the previous ordination (10%) suggests greater change in species composition with the longer recovery time. Even at this longer time scale, the majority of forbs and grasses still scored positively on the OMI *X*-axis, indicating higher abundance on burned areas, while all cacti again had negative scores. However, 42% of woody species scored positively on the OMI *X*-axis for the long-term dataset, compared with only 12% in the short-term dataset.

### Identification of significant traits

**Figure 3:**
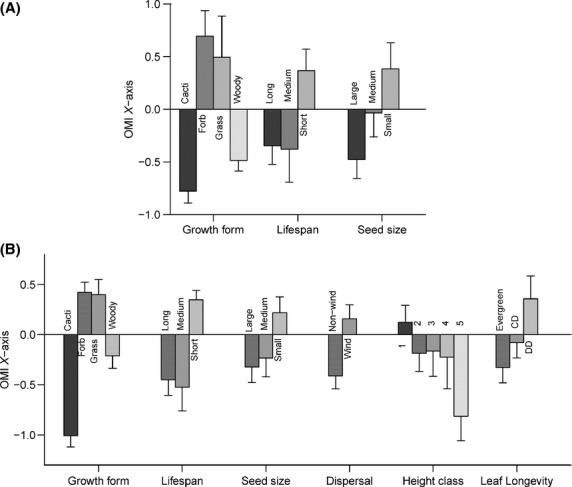
Bar plots showing mean species scores (+ SE) for different levels of life-history traits on OMI *X*-axis (corresponding to fire treatment) for the short (A) and long-term (B) datasets. Higher scores on this ordination axis indicate greater abundance in burned areas relative to unburned areas. Only traits found to exert a significant influence on species scores from each dataset are shown.

Among the full set of eight traits, six explained a significant amount of variability in species' OMI axis scores from either the short- or long-term datasets, based on perMANOVA (Table 3). Growth form explained the most variability in OMI species scores across both datasets, while lifespan and seed size were also significant at both time scales. Vegetative spread and growth structure were not significant in any test and did not appear to influence species responses to fire. A comparison of mean species scores for the different levels of each significant trait on the OMI *X*-axis (Fig. 3A and B) indicates that wildfire favored species with short lifespans, small seed size, wind-dispersed seeds, short heights, and deciduous leaves. As previously noted, the forb and grass growth forms were also the most favored by fire, while woody species as a whole and cacti were reduced in burned areas relative to unburned controls.

**Table 3:** Results from perMANOVA testing of species traits against species scores derived through outlying mean index (OMI) ordinations of both short- and long-term datasets of burned Mojave Desert vegetation. Except where noted, tests were conducted separately for all species and for woody species alone (after forward slash). All trait codes follow Table 2. Significant (*P* ≤ 0.05) test results are displayed in bold.

Trait	Short-term		Long-term	
	*R*^2^	*P*	*R*^2^	*P*
Growth form^1^	0.42	<**0.01**	0.31	**<0.01**
Growth structure^1^	0.08	0.18	0.05	0.26
Height	0.08/0.05	0.08/0.38	0.07/0.10	**0.04**/**0.04**
Leaf longevity^2^	NA^3^/0.18	NA*/*0.06	NA/0.14	NA**/0.05**
Lifespan	0.17/0.15	**0.02**/0.33	0.21/0.31	<**0.01**/<**0.01**
Seed dispersal	0.01/0.01	>0.50/>0.5	0.11/0.08	<**0.01**/0.09
Seed size	0.15/0.08	**0.02**/>0.5	0.13/0.10	**0.01**/**0.04**
Vegetative spread	0.07/0.03	0.29/0.37	0.04/0.02	0.43/0.38

1 Tested for all species.

2 Tested for woody species alone.

3 NA = not applicable.

Several differences emerged when comparing results across datasets, particularly for the tests of woody species. Most notably, none of the traits had a significant influence on woody species' responses to fire in the short-term dataset, whereas four traits were significant when the long-term dataset was considered, including lifespan, leaf longevity, seed size, and height (Table 3; Fig. 3B). This difference in test results was likely because a greater number of woody species were associated with burned areas in the long-term dataset (42% scoring positively on the OMI *X*-axis) than for the short-term (12% scoring positively on the OMI *X*-axis), indicating that some woody species were able to increase abundance in burned relative to unburned areas over this longer time scale. However, sample size may also have played a role in the test results, as there were only 16 woody species in the short-term dataset compared with 31 in the long-term dataset.

### Derivation and evaluation of functional groups

#### Classification of woody species

**Figure 4:**
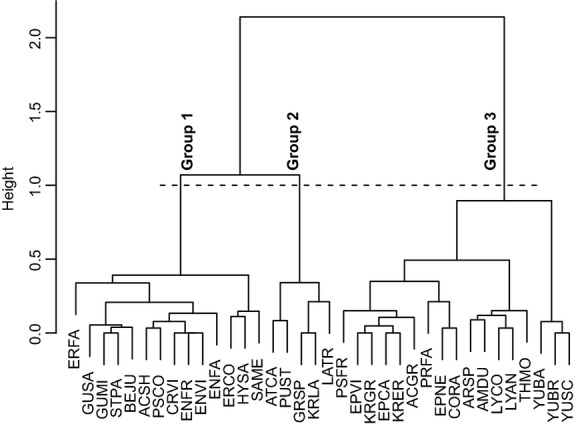
Dendrogram from hierarchical agglomerative cluster analysis of 36 woody species found in the Mojave Desert. Five traits were used as variables in the clustering, including height, lifespan, seed size, seed dispersal, and leaf longevity. Three functional groups were chosen by pruning the dendrogram at a height of approximately 1 (dashed line). Translation of species codes is provided in Appendix S1.

We included five traits in the hierarchical agglomerative cluster analysis that were significant (*P* ≤ 0.05) or marginally significant (*P* < 0.1) for this growth form in perMANOVA testing of either the short- or long-term datasets, including: lifespan, seed size, seed dispersal, height, and leaf longevity. We pruned the dendrogram created through cluster analysis at a height of approximately 1.0, resulting in three potential PFTs (Fig. 4). This cut point corresponded to the lowest branch in the dendrogram that sill retained adequate replication within each group and also reflected fundamental life-history tradeoffs. Woody species in Group 1 were characterized by short lifespans, small, wind-dispersed seeds, an average height class of 2.1, and drought-deciduous leaves (Table 4) – traits that were all favored by fire (Fig. 3A and B). In contrast, woody species in Group 3 were characterized by long lifespans and large, nonwind-dispersed seeds, an average height class of 3.3, and either evergreen or deciduous leaves (Table 4) – many traits that were not favored by fire (Fig. 3A and B). Species in Group 2 possessed traits making them intermediate between Groups 1 and 3, including moderate to long lifespans, medium-sized, wind-dispersed seeds, evergreen leaves, and an average height class of 3.4 (Table 4).

**Table 4:** Characteristics of PFTs identified through hierarchical agglomerative cluster analysis of 36 woody perennial species occurring on burned or unburned control transects in the Mojave Desert. Typical trait values for each functional group are provided, as defined in Table 1.

Group	Lifespan	Seed mass	Dispersal	Height classes	Leaf longevity
1	Short	Small-Medium	Wind	1–3 (2.1)^1^	DD
2	Moderate-Long	Medium	Wind	2–5 (3.4)	Evergreen
3	Long	Medium-Large	Non-wind	2–6 (3.3)	DD-Evergreen

1 Average height class for all species within each group denoted in parentheses.

#### Evaluation of functional groups

**Figure 5:**
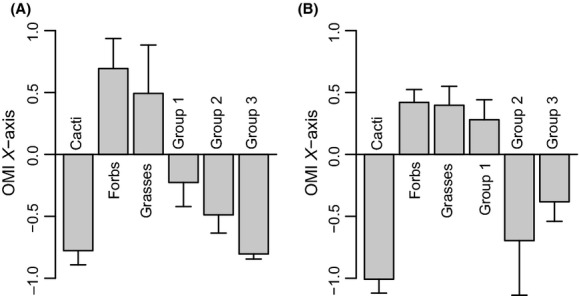
Response of different growth forms and functional groups to wildfire in the Mojave Desert. Panels show mean scores (+ SE) for species in different functional groups on OMI X-axes for the (A) short- and (B) long-term datasets, corresponding to fire treatment.

Functional group was a significant predictor of OMI species scores in perMANOVA models for both the short-term (*R*^2^ = 0.55, *P* = 0.0001) and the long-term (*R*^2^ = 0.49, *P* = 0.0001) datasets. For the short-term dataset, the functional group model explained 13% more variability than growth form, the most predictive individual trait (*R*^2^ = 0.42; Table 2). All three woody species groups had negative overall means on the short-term OMI *X*-axis, although species in Group 1 had the highest average scores (Fig. 5A). For the long-term dataset, the functional group model was able to explain 18% more variability in species scores than growth form (*R*^2^ = 0.49 versus 0.31). Woody species in Group 1 had a mean (± SE) of 0.28 (± 0.16) on the OMI *X*-axis for the long-term dataset, indicating greater abundance in burned areas (Fig. 5B). This differed from woody species in Groups 2 and 3, which had negative means on the OMI *X*-axis (−0.69 ± 0.44 and −0.38 ± 0.16, respectively). Cacti had the lowest overall mean on the OMI *X*-axis for both the short- and long-term datasets, while forbs and grasses had the highest mean scores across both datasets (Fig. 5A and B).

## Discussion

Burned areas in the Mojave Desert were clearly differentiated from unburned areas in terms of the life-history traits of their component perennial species, particularly with respect to growth form. Additionally, woody species could be grouped into PFTs with a consistent response to fire based on recurring combinations of life-history traits. This consistency in grouping occurred because traits that were decreased by fire (e.g., long lifespan, large seeds, tall height) combined in some species, while traits that were increased (short lifespan, small wind-dispersed seeds, deciduous leaves, low height) combined in others (Table 4). Of particular note is that widespread, dominant species in this ecoregion, including *Larrea tridentata*, *Coleogyne ramosissima*, and *Yucca brevifolia*, fell within Groups 2 and 3 in our analysis and possess a set of traits limiting their ability to regenerate following fire. Given the scope of our datasets, which spanned different vegetation communities, temporal scales, and a broad geographic region, our results suggest that a PFT approach based on simple morphological traits can be used to model the effects of fire across the Mojave Desert.

Among the morphological traits we considered, growth form had the largest influence at both time scales of our analysis (Table 3). Indeed, this trait alone could serve as a general predictor for the short-term (1–4 years) responses of most species to fire. Forbs and grasses were consistently more abundant in burned areas across both time scales of our analysis, whereas cacti were consistently reduced by fire. Only woody species exhibited a variable response that warranted classification into separate PFTs. The predictive value of growth form as a disturbance-response trait likely results from its influence on a variety of factors, including resistance and resilience to physical damage, growth rate, and generation time (Lavorel et al. 2007). For example, cacti are typically fire-killed due to tissue desiccation (Esque et al. 2004), produce large seeds with limited dispersal ability, and have episodic recruitment patterns with narrow climatic requirements for successful seedling establishment (Godínez-Alvarez et al. 2003). Several studies have suggested that PFT approaches should be hierarchically structured within major growth forms (Lavorel et al. 1997; Pausas and Lavorel 2003), and this assertion is supported by our analysis. Although growth form has high predictive value as a trait, the specific responses we detected may not be generalizable to other fire-prone environments or disturbance types. For example, woody species in fire-prone Mediterranean shrublands are able to regenerate shortly after fire through both seeding and resprouting (Keeley et al. 2006), in stark contrast to Mojave Desert species.

On average, the fire responses of PFT groups we identified for woody species were consistent with predictions based on life-history theory and with the IFC model. For example, woody species in Group 3 were characterized by long lifespans and large seeds, traits that reflect a slow life-history with greater investment toward individual survival (Grime 1977; Adler et al. 2014). In contrast, Group 1 species had small, wind-dispersed seeds, short lifespans, and deciduous leaves – traits thought to reflect investments in fecundity and growth (Franco and Silvertown 2004; Adler et al. 2014). Species in Group 2, though low in number (*n* = 5), were intermediate, with some traits linked to investments in fecundity (medium-sized, wind-dispersed seeds) and others linked to investment in survival (evergreen leaves, long lifespan; Adler et al. 2014). Life-history theory suggests that investments in growth and fecundity increase a species ability to respond to disturbance (Grime 1977), and thus our results (Group 1 species were favored by fire, Group 2 and 3 species were reduced) are largely consistent with this prediction. Our finding that species with greater longevity were slowest to recover is also consistent with the IFC model, which predicts that differences in species longevities will shape successional trajectories (Egler 1954; Wilson et al. 1992).

It is also important to consider ecological mechanisms beyond links to life-history through which the traits we considered may have influenced species responses to fire. A variety of interrelated factors influence the “regeneration niche” in arid environments, or the ability of species to regenerate via seed. Large seeds are typically rare in desert seed banks (Guo et al. 1998; DeFalco et al. 2009), in part due to preferential granivory by ants and rodents (Price and Joyner 1997; Hulme 1998). Long-lived desert species with large seeds – including cacti - typically do not have persistent soil-stored seed banks and instead produce copious quantities of seed during rare, favorable climatic events (Goldberg and Turner 1986; Cody 2000; Godínez-Alvarez et al. 2003; Chesson et al. 2004; Reynolds et al. 2012). Much of the reproductive potential for these species is therefore stored in aboveground canopies, which may be eliminated by a single fire. In contrast, small seeds compose a large fraction of desert seed banks (Guo et al. 1999) and are able to sift deeper into the soil and survive peak fire temperatures (Brooks 2002; Esque et al. 2010). Short-lived and small-seeded desert species also produce seedlings more frequently than do long-lived species, indicating less exacting climatic requirements for germination (Goldberg and Turner 1986). Aided by the potential for long-distance wind dispersal, small-seeded species probably have broader regeneration niches following fire than large-seeded species due to both life-history and other ecological mechanisms.

Interestingly, we found no support for an influence of vegetative spread capability on woody species response to fire in either dataset. This, along with the finding that only Group 1 species were favored by fire even at a longer time scale, appears to support our assertion that resprouting is less important as a regeneration mechanism than seedling establishment due to low and variable precipitation levels (Clarke et al. 2005; Abella 2009; DeFalco et al. 2010). Also, resprouters tend to be long-lived, with heavy propagules – characteristic of the fire-reduced Groups 2 and 3 in our analysis (Pausas et al. 2004). A lack of effective resprouting would distinguish Mojave Desert vegetation from other fire-prone communities where resprouting is a common fire-response syndrome, such as Mediterranean shrublands (Allen 2008). However, it should be noted that a small number of species in PFT Group 3 were associated with burned areas in the long-term dataset, including *Ephedra viridis*, *Prunus fasciculata*, *Yucca baccata*, and *Lycium cooperi*, and it is possible that these species were able to regenerate through low levels of resprouting. However, unburned patches can also occur during low-intensity surface fires in the Mojave Desert, where individual plants may survive with only slight scorching (Abella 2009).

Several caveats to our analysis should also be recognized. First, we were unable to account for intraspecific variability in species traits, which may be as important as interspecific variability in influencing species responses to fire (Dantas et al. 2012). Secondly, some traits had to be represented through ordinal classes (e.g., height, seed size) rather than with a continuous approach because traits for many species in the Mojave Desert have not been quantitatively measured across a variety of conditions. Both of these limitations may have reduced the accuracy of our PFT approach with respect to our study system, while the second may reduce its generality to other contexts. We also were unable to incorporate information concerning such factors as fire intensity, fire season, topography, and postfire climatic conditions when developing our PFT model, although variation in these factors could conceivably alter the way in which traits influence species' responses to fire. For example, wildfires in the Mojave Desert may range from surface to crown fires depending on annual fuel buildup (Brooks and Minnich 2006), and this variation in fire intensity could change how woody species of different heights are affected. Similarly, postfire climatic conditions may influence survival rates of scorched vegetation and newly emerged seedlings (DeFalco et al. 2010). Despite these limitations, our PFT approach was able to predict how the majority of species responded to fire, indicating its generality to a wide range of contexts.

Fire is becoming a recurrent disturbance in the Mojave Desert driven by an invasive grass/fire feedback cycle (D'Antonio and Vitousek 1992; Brooks and Esque 2002; Brooks et al. 2004), with once-burned areas becoming more likely to reburn (Brooks and Matchett 2006). Changes to regional climates expected over the next century may exacerbate this cycle, particularly more frequent shifts between moist El Niño climatic phases that promote annual fuel buildup and subsequent dry La Niña phases that promote fire (McCabe et al. 2004; Allen and Soden 2008). Recurrent fires may reduce or eliminate the dominant vegetation strata in this ecosystem through trait convergence, a process that could have cascading effects across trophic levels (Brooks and Esque 2002; Esque et al. 2003; Vamstad and Rotenberry 2010).

## Supporting Information

**Appendix S1.** Species Scientific Names and Traits. **Table S1.** Species names, species codes, and trait values for all species included in the study.
